# The Development of Eupyrene Sperm Is Dependent on Sperm-Leucylaminopeptidase in *Bombyx mori*

**DOI:** 10.3390/insects17040389

**Published:** 2026-04-03

**Authors:** Hongxia Kang, Guan Man, Yutong Liu, Anjiang Tan, Kai Chen

**Affiliations:** 1 Jiangsu Key Laboratory of Sericultural and Animal Biotechnology, School of Biotechnology, Jiangsu University of Science and Technology, Zhenjiang 212100, China; 18139739068@163.com (H.K.); mg073100@163.com (G.M.); liuyutong010203@163.com (Y.L.); atan@just.edu.cn (A.T.); 2 Key Laboratory of Silkworm and Mulberry Genetic Improvement, Ministry of Agriculture and Rural Affairs, Sericultural Scientific Research Center, Chinese Academy of Agricultural Sciences, Zhenjiang 212100, China

**Keywords:** *Bombyx mori*, Leucylaminopeptidases, fertility, dimorphic spermatogenesis

## Abstract

Sexual reproduction is the predominant mode of reproduction in animals, and spermatogenesis is the fundamental step in this process. As the model organism for lepidopteran, the silkworm *Bombyx mori* exhibits typical dichotomous spermatogenesis, producing both nucleated (eupyrene) and anucleate (apyrene) sperm. Leucylaminopeptidases (LAPs), members of the M17 metalloprotease family, are characterized by their ability to cleave leucine residues from the N-terminus of peptides. In addition to this canonical function, they have been implicated in male fertility in mammals and *Diptera*. Nevertheless, whether LAPs are required for dimorphic spermatogenesis in Lepidoptera remains to be clarified. Here, we demonstrated that *Sperm-Leucylaminopeptidase* (*S-LAP*) plays vital roles in the silkworm eupyrene sperm development. Similar to the testis-specific expression pattern of eight *S-LAPs* in *Drosophila melanogaster*, *BmS-LAP* was also predominantly expressed in testis. Depletion of *BmS-LAP* via CRISPR/Cas9 system resulted in male sterility, while the fertility of female mutant was unaffected. Notably, male mutants displayed severe defects in the formation and migration of eupyrene sperm, whereas apyrene sperm development appeared normal. In addition, RNA-seq and qRT-PCR analyses demonstrated that spermatogenesis defects were associated with energy metabolism and flagellar assembly. Our study provides the first evidence that LAP is necessary for dimorphic spermatogenesis in *Lepidopteran*, offering new insights into the molecular basis of male infertility.

## Simple Summary

While the role of leucine aminopeptidase (LAP) in reproductive development is well-established in mammals and Diptera, its function in the male reproductive system of lepidopteran insects remains largely unexplored. In this study, we performed functional analysis of *Bombyx mori sperm-Leucineaminopeptidase* (*BmS-LAP*), the sole homolog of *Drosophila melanogaster S-LAPs* in *B. mori*, using CRISPR/Cas9 system. Depletion of *BmS-LAP* resulted in complete male sterility while the fertility of female silkworms remained unaffected. The male sterility phenotype was caused by developmental defects of eupyrene sperm, visualized as nuclear mislocalization and a failure of flagellar elongation. Furthermore, morphological observation and double copulation assays revealed that the *BmS-LAP* mutation had no significant effect on apyrene sperm development. This study provided the first evidence that LAP is implicated in dimorphic spermatogenesis.

## 1. Introduction

Spermatogenesis is a highly intricate fundamental process for sexual reproduction in animals and entails precisely orchestrated series of events involving mitotic proliferation, meiotic division, and spermatogenic differentiation [1,2]. In contrast to the canonical spermatogenesis in most species, some exhibit sperm polymorphism, where an individual male regularly produces multiple distinct types of sperm [3,4,5]. Lepidopteran species exhibit unique dichotomous spermatogenesis, generating coexisting nucleated (eupyrene) sperm responsible for fertilization, and anucleated (apyrene) sperm believed to play auxiliary roles in sperm competition and migration [1,6,7,8,9]. Despite a long history of study on dimorphic spermatogenesis, the mechanisms underlying the differentiation of the two sperm types remain poorly understood. The silkworm, *Bombyx mori*, is an important economic insect and serves as an ideal model lepidopteran organism, owing to its well-annotated genome and well-established genetic manipulation technologies [10,11,12,13,14]. *B. mori* exhibits typical dimorphic sperm like other Lepidoptera. The maturation of eupyrene and apyrene sperm follows distinct developmental timelines and involves dramatic cellular transformations, including marked nuclear condensation, active flagellar elongation, and extensive cytoplasmic remodeling [15,16,17]. The mature eupyrene sperm bundle is characterized by an elongated nucleus with needle-shaped chromatin and a long tail [18,19]. Its final maturation involves the elimination of residual cytoplasm via “peristaltic squeezing” [19]. In contrast, apyrene sperm bundles are shorter [6,20]. Similarly, a peristaltic squeezing process discards their round, centrally located nuclei, resulting in the mature anucleated sperm bundle [20,21,22].

As exopeptidases belonging to the M17 metalloprotease family, leucine aminopeptidases (LAPs) are characterized for their preferred cleavage of N-terminal leucine but also exhibit activity toward additional hydrophobic amino acids, including phenylalanine and isoleucine [23,24,25]. The active form of LAP is a hexamer, each subunit of which contains approximately 500 amino acid residues [26]. Its active site is characterized by a binuclear metal center, typically containing two non-equivalent metal ions [27,28,29,30]. In addition to their well-established enzymatic function, LAPs are implicated in biological processes such as the regulation of male fertility [31]. Several studies have shown that LAPs are highly expressed in the seminal plasma of mammals, and their activity is closely linked to male fecundity [32].

LAPs are also reported to be involved in male fertility in Diptera such as *Drosophila melanogaster* and *Aedes aegypti* [15,33]. The structural components of insect sperm are very similar to those of mammalian sperm, sharing many common features such as the plasma membrane, acrosome, elongated nucleus, axoneme, and mitochondria [34,35,36,37,38,39,40]. In insect sperm, mitochondria are typically located in the midpiece, arranged around the flagellar axoneme [19,41,42]. During spermatogenesis, mitochondria undergo significant reorganization to form two kinds of mitochondrial derivatives in mature sperm [43,44]. In *D. melanogaster* sperm, one of the mitochondrial derivatives accumulates paracrystalline material that is crucial for spermatogenesis [45,46]. The main components of this material are eight testis-specific leucine aminopeptidases (LAPs), which are consequently designated Sperm-Leucylaminopeptidases 1-8 (S-LAP1-8). Each S-LAP is essential for spermatogenesis, and they perform non-redundant functions [33]. In *Aedes aegypti*, LAP1 also contributes to fertility. It localizes to sperm mitochondria, and *LAP1* mutants exhibit developmental defects in the mitochondrial derivatives along with autophagy, which impairs spermatogenesis and ultimately leads to male sterility. Additionally, female *LAP1* mutants lacking show significant abnormalities in ovarian and follicular development [15]. In the lepidopteran model insect, *Bombyx mori*, defects in mitochondrial derivatives also lead to male infertility [8]. Nevertheless, whether *LAP* is implicated in dimorphic spermatogenesis of *B. mori* remains to be clarified.

In the current study, we performed functional analysis of *BmS-LAP*, the sole homolog of *D. melanogaster S-LAPs* in *B. mori*, using a binary transgenic CRISPR/Cas9 system. Our data revealed that *BmS-LAP* was specifically expressed in testis, and depletion of *BmS-LAP* resulted in complete male sterility due to developmental defects of eupyrene sperm, visualized as nuclear mislocalization and a failure of flagellar elongation. Subsequent RNA-seq and qRT-PCR analysis indicated significant disruptions in pathways related to energy metabolism, glycosylation, and intraflagellar transport. This study establishes *BmS-LAP* as a critical factor for eupyrene sperm development in silkworms and provides new mechanistic insights into the molecular regulation of dichotomous spermatogenesis in Lepidoptera.

## 2. Materials and Methods

### 2.1. Silkworm Strain

All experiments in this study were conducted using the multivoltine, nondiapausing silkworm strain Nistari. Larvae were reared on fresh mulberry leaves under standard rearing conditions at 25 °C [14,47,48].

### 2.2. CRISPR/Cas9-Mediated Construction of Mutants

In this study, a binary transgenic CRISPR/Cas9 system was employed to construct the *ΔBmS-LAP* mutant. The transgenic line nos-Cas9 (*IE1-EGFP-nos-Cas9*) expressing the Cas9 protein had been established in previous studies [43,44]. The transgenic line U6-sgRNA (*IE1-DsRed-U6*-sgRNA1-*U6*-sgRNA2) was designed to drive the expression of two single-guide RNAs (sgRNAs) targeting the *BmS-LAP* gene under the control of the U6 promoter. The sgRNA sequences and primer information are listed in Table S1. A mixture of the transgenic plasmid, helper plasmid, and piggyBac transposon mRNA was microinjected into 640 G0 embryos. G0 moths were crossed with wild-type moths to generate G1 broods. Individuals marked with red fluorescence among the G1 progeny were screened using a fluorescence microscope (AZ100, Nikon, Tokyo, Japan). The two silkworm lines expressing sgRNAs and Cas9 protein were crossed to generate F1 progeny, and mutant lines co-expressing GFP and RFP were screened using fluorescence microscopy.

### 2.3. Mutagenesis Analysis

Genomic DNA was isolated from larval-stage mutants using the standard SDS lysis-phenol extraction method. The samples were incubated with proteinase K at 56 °C for 5 h, followed by RNase treatment and purification. Specific primers flanking the target site were designed, and PCR amplification was carried out with 100 ng of genomic DNA as the template. The resulting PCR products were then purified, cloned into the pJET1.2 vector, and sequenced.

### 2.4. RNA Isolation and qRT-PCR

Total RNA was isolated from individual silkworm tissues collected at three developmental stages—day 0 of the fifth instar (L5D0), day 3 of the fifth instar (L5D3), and the wandering stage (W)—using TRIzol reagent (Invitrogen, Waltham, MA, USA). The extracted RNA was then reverse transcribed into cDNA employing an RT kit equipped with gDNA Eraser (Takara, Kusatsu, Japan). Expression levels of target genes at the mRNA level were assessed via SYBR Green–based real-time quantitative PCR, with the ribosomal protein gene BmRP49 serving as the internal control for data normalization. Quantification was carried out across three independent biological replicates, each containing three technical replicates. The primers utilized for qRT-PCR are provided in Table S1.

### 2.5. Fluorescent Staining of Sperm Bundles

Sperm bundles, as well as sperm at various developmental stages (fifth instar larval and pupal stages), were collected into 1.5 mL centrifuge tubes and fixed in fixative solution for 2 h for immunostaining. After three washes with PBS, each lasting 5 min, the samples were incubated with TRITC-labeled phalloidin for 1 h to visualize actin, followed by Hoechst staining for 10 min to label nuclei. Following three additional PBS washes, the samples were mounted onto glass slides and examined using a Nikon C2 confocal microscope (Nikon, Tokyo, Japan).

### 2.6. Paraffin Section and Hematoxylin Eosin Staining

Testes from both mutants and wild-type individuals were dissected and promptly fixed in a fixative solution consisting of anhydrous ethanol, chloroform and acetic acid, in a ratio of 6: 3: 1 (*v*/*v*/*v*) for 24 h. Following fixation, the samples were transferred to 70% (*v*/*v*) ethanol for storage, subsequently dehydrated three times with anhydrous ethanol, and cleared three times with xylene. The tissues were then embedded in paraffin overnight. Sections of 5 μm thickness were prepared using a Leica microtome (RM2235, Leica, Wetzlar, Germany). After deparaffinization and rehydration, the sections were stained with a hematoxylin and eosin mixture for histological examination. Images were captured and analyzed using an Olympus BX53 microscope (Olympus, Tokyo, Japan).

### 2.7. Statistical Analysis

All data were analyzed by GraphPad Prism (version 5.01) and presented as ±SEM. The statistically significant differences were measured by Student’s *t*-test with a paired, 2-tailed distribution (*, *p* < 0.05; **, *p* < 0.01; ***, *p* < 0.001).

## 3. Results

### 3.1. Expression Pattern of BmS-LAP and Mutant Construction

**Figure 1:**
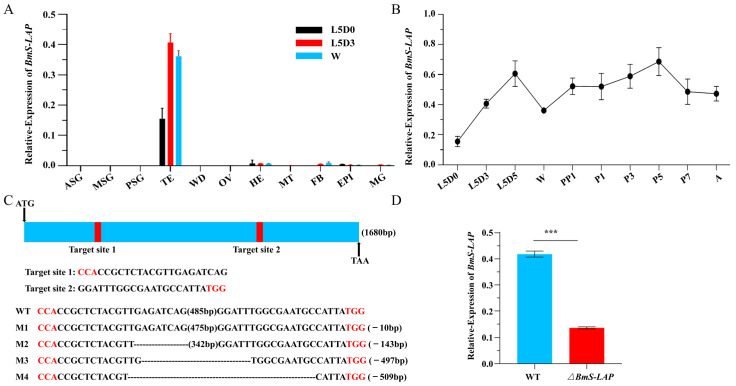
**Expression pattern and mutant construction of *BmS-LAP*.** (**A**) Relative mRNA levels of *BmS-LAP* in eleven tissues at three different stages: newly molted larvae at the fifth larval stage (L5D0), Third day of the fifth larval stage (L5D3), wandering stage (W). Tissues tested were Anterior silk gland (ASG), Middle silk gland (MSG), Posterior silk gland (PSG), testis (TE), wing disc (WD), ovary (OV), head (HE) and malpighian tubule (MT), fat body (FB), epidermis (EPI), midgut (MG). (**B**) Relative expression levels of *BmS-LAP* in the testes at different developmental stages. (**C**) Mutations induced by CRISPR/Cas9 system. The sequence of the wildtype is displayed at the top. The dotted lines indicate the deleted residues, the PAM sequence is in red, the amount of the nucleotides deleted are shown at the right. (**D**) The mRNA expression level of *BmS-LAP* in three individual WT and mutant testes at wandering stage. The asterisks (***) indicate the significant differences (*p* < 0.001) relative to WT.

According to the previous study, all the eight S-LAP proteins exhibit testis-specific expression pattern in *D. melanogaster* [33,49]. Similarly, qRT-PCR analysis showed that *BmS-LAP* was predominantly expressed in testis at all developmental stages tested (Figure 1A). In addition, the relative expression of *BmS-LAP* in testis increased progressively from early to late larval stages and was sustained at high levels during pupal and adult stages, suggesting that the gene might be essential for testis development (Figure 1B).

To determine whether the gene plays a role in testis development and spermatogenesis, we generated *BmS-LAP* mutants using a previous described binary CRISPR/Cas9 system [11,12,45]. In short, two silkworm lines were constructed, one expressing two sgRNAs targeting the single exon of *BmS-LAP* gene was marked with EGFP, while the other one generating Cas9 protein was labeled with DsRed2. Genomic PCR followed by Sanger sequencing confirmed mutations in the *BmS-LAP* gene locus of the heterozygous F1 progeny (*ΔBmS-LAP*) derived from the two lines (Figure 1C). Furthermore, qRT-PCR analysis demonstrated significantly reduced *BmS-LAP* transcript levels in testis of *ΔBmS-LAP* individuals, confirming the establishment of *BmS-LAP* mutants (Figure 1D).

### 3.2. Depletion of BmS-LAP Leads to Male Sterility

**Figure 2:**
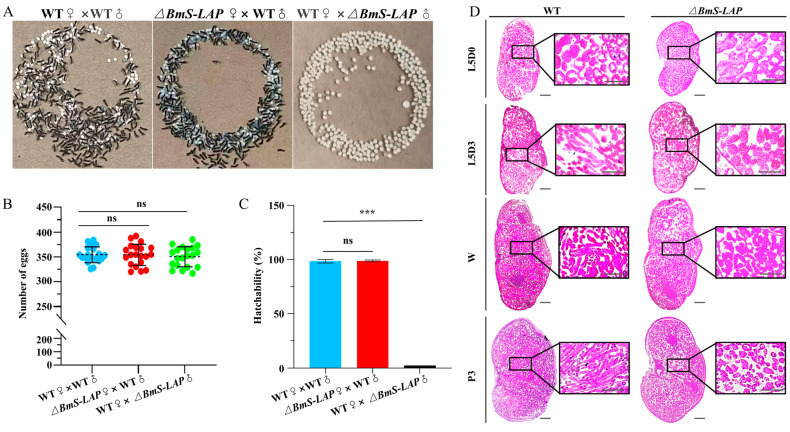
***BmS-LAP* deficiency results in male sterility.** (**A**) Photographs of eggs after 10 days laid by wildtype females mated with wildtype or mutant males. (**B**) Number of eggs laid by female. Data are mean ± SEM (*n* = 20). The dashed line indicates the average value. (**C**) Hatchability of eggs laid by females in different groups (*n* = 20). (**D**) Morphologies of internal structure of wildtype (**left**) and *BmS-LAP* mutant (**right**) testis. Scale bar: 200 μm. “ns” represents non-significant difference; “***” represents *p* < 0.001.

*BmS-LAP* mutants developed normally throughout all developmental stages, with no obvious deleterious phenotypes observed. However, further analysis demonstrated that male *BmS-LAP* mutants were completely sterile while the fertility of female mutants were unaffected. Although WT females mated with mutant males laid a comparable number of eggs to the control group (WT × WT), none of progeny derived from *ΔBmS-LAP* males hatched (Figure 2A–C).

To clarify the underlying cause of the sterility phenotype, we performed paraffin-embedded tissue sections followed by hematoxylins and eosin staining for WT and mutant testis at diverse developmental stages. Although the morphology of the mutant testes appeared normal compared with that of WT silkworms, the process of spermatogenesis in mutants was gradually arrested. The development of two kinds of sperm follows distinct developmental timelines, and eupyrene sperm bundles undergo morphological changes from early fifth larval instar, while apyrene sperm bundles start to elongate from the wandering stage [50]. At early larval stage (newly molted larvae at fifth larval stage, L5D0), no significant difference was observed between WT and mutant testis. At later developmental stages, however, the mutant phenotype became apparent. On the third day of the fifth larval stage (L5D3), there are numerous elongated eupyrene sperm bundles in WT testis; by contrast, only round cysts and short sperm bundles were detected in mutants. Furthermore, the phenotype was more apparent at wandering stage (W) and the third day of pupal stage (P3) (Figure 2D).

### 3.3. BmS-LAP Mutation Impaired the Formation of Eupyrene Sperm

**Figure 3:**
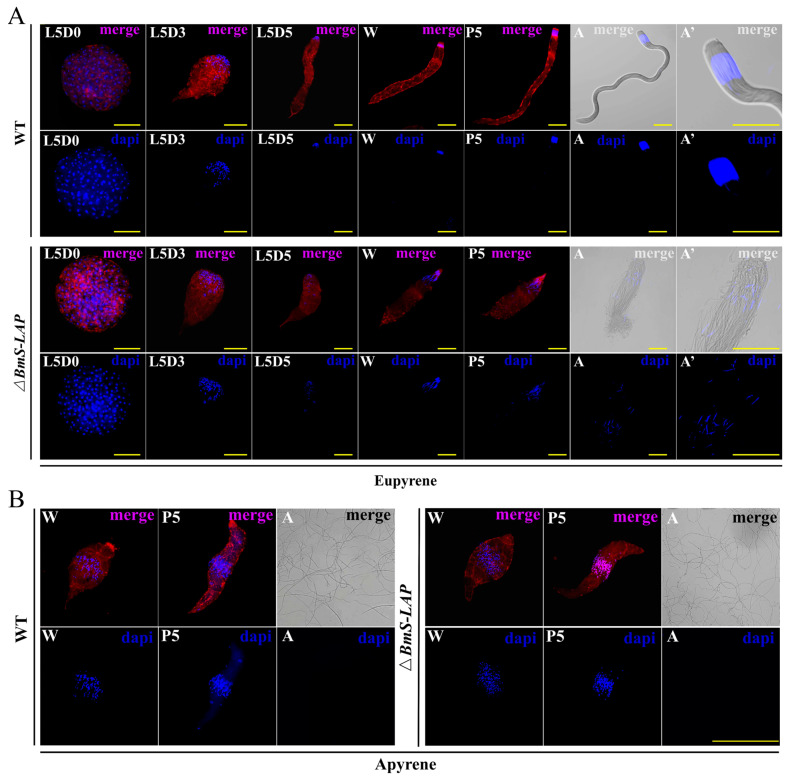
***BmS-LAP* mutation impaired the formation of eupyrene sperm.** (**A**) The eupyrene sperm bundles showed abnormal location, spacing of nuclei and shorter flagella in *ΔBmS-LAP* males. Fluorescence image of eupyrene and apyrene sperm bundles in testes of wildtype (WT) and *ΔBmS-LAP* males from L5D0 to adult stage. A’ represents the enlarged view of the adult stage image. (**B**) The morphology of anucleate sperm in the mutant (*ΔBmS-LAP*) showed no significant difference compared to that in the wild type (WT). W: wandering stage; P5: fifth day of pupal stage; A: adult stage. Scale bar: 100 μm.

To further characterize the male sterility phenotype, we performed fluorescence staining for sperm bundles at different developmental stages. In the WT testis, eupyrene sperm began elongating from stage L5D0, with their nuclei gradually condensing and aggregating at the bundle head. As the result, the mature eupyrene sperm bundle in adults appeared as a long tail, capped by a rounded head tightly packed with needle-shaped nuclei. By contrast, while elongation of eupyrene sperm was observed to initiate in the early larval stage, the process was gradually disrupted and ultimately arrested. Compared to the WT, the mutant eupyrene sperm bundles were significantly smaller and exhibited abnormal morphology. Furthermore, nuclei condensation and aggregation were also impaired in the mutant eupyrene sperm bundles. Consequently, the mutant eupyrene sperm bundles in adults exhibited a collapsed architecture, characterized by shortened tails, dispersed nuclei, and a loss of structural coherence (Figure 3A).

By comparison, no discernible defects were observed in the development of mutant apyrene sperm. The process initiated normally with bundle elongation at the wandering stage and proceeded through proper nuclei elimination and individualization during maturation. In summary, our results establish that *BmS-LAP* is crucial for eupyrene sperm development, but dispensable for apyrene sperm formation (Figure 3B).

### 3.4. Migration of Mutant Eupyrene Sperm Was Disrupted

**Figure 4:**
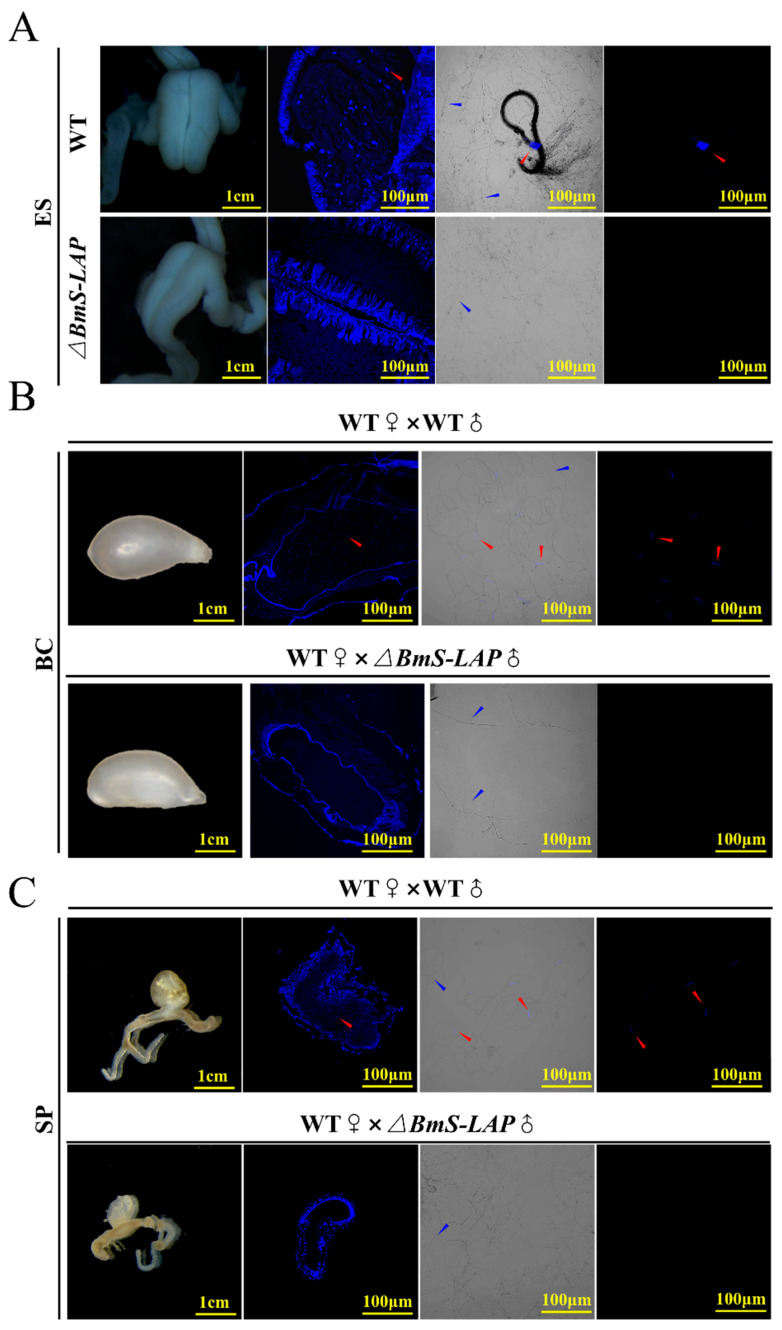
**Migration of mutant eupyrene sperm was disrupted.** (**A**) Morphologies of internal structure of ejaculatory seminalis (ES) of unmated WT and *ΔBmS-LAP* males. (**B**) Morphologies and internal structure of bursa copulatrix (BC) in WT females mated with WT and *ΔBmS-LAP* males. (**C**) Morphologies and internal structure of spermatheca (SP) in WT females mated with WT and *ΔBmS-LAP* males. Red arrows indicate eupyrene sperms, and blue arrows indicate apyrene sperms. Paraffin-embedded sections were stained with Hoechst 33258. For each row, column 1 is a bright-field image of the organ; column 2 is a paraffin section of the organ stained with Hoechst; column 3 is the merge of bright-field and 405 nm channels after Hoechst staining; and column 4 is the Hoechst-stained organ imaged under 405 nm alone.

After maturation, spermatozoa are released from the testes via spermiation and are subsequently transported to the male genital ducts [18]. Initially, apyrene sperm bundles dissociate into individual spermatozoa within the vas deferens, while eupyrene sperm remain bundled and are transported alongside them. Both types are ultimately conveyed to the ejaculatory seminalis [51,52]. Upon copulation, they are transferred to the female’s bursa copulatrix. Subsequently, the sperm migrate to and are stored in the spermatheca, where they ultimately fertilize the eggs during oviposition. To better show the defects of eupyrene sperm bundles, we examined the migration of sperms in male and female genital tract. While both individualized apyrene spermatozoa and eupyrene sperm bundles were present in WT ejaculatory seminalis, only individualized apyrene spermatozoa were detected in *ΔBmS-LAP* mutants (Figure 4A). After migration from the male ejaculatory seminalis to the female bursa copulatrix during copulation, eupyrene sperm bundles typically dissociate into individual spermatozoa [53]. To track this process, we examined the bursa copulatrix and spermathecae of WT females mated with WT or *ΔBmS-LAP* males. While both individual spermatozoa types are found in the bursa copulatrix and spermathecae of WT females mated with WT males, only apyrene spermatozoa are present in these organs when females are mated with *ΔBmS-LAP* males (Figure 4B,C). The results indicate that *ΔBmS-LAP* specifically impairs eupyrene sperm migration, with no observed effect on the migration of apyrene sperm.

**Figure 5:**
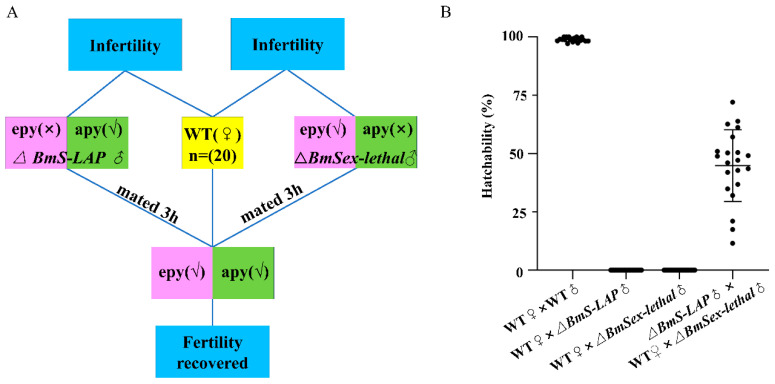
Double mating partially restores fertility of *BmS-LAP* mutant males. (**A**) Schematic diagram of the double mating assay. (**B**) Hatchability of eggs laid by female of the double mating assay was evaluated based on the ratio of fertile individuals to the total experimental population (*n* = 20).

To further validate whether apyrene sperm function is compromised in *BmS-LAP* mutants, we conducted double copulation assays using male *BmSxl* mutants, which possess abnormal apyrene sperm but normal eupyrene sperm [8]. WT females produced no offspring when mated individually with *BmS-LAP* or *BmSxl* mutant males. Nevertheless, partial fertility was restored through successive mating with both mutants, demonstrating the functional competence of apyrene sperm from *BmS-LAP* mutants (Figure 5).

### 3.5. Transcriptomic Analyses of BmS-LAP Mutants

**Figure 6:**
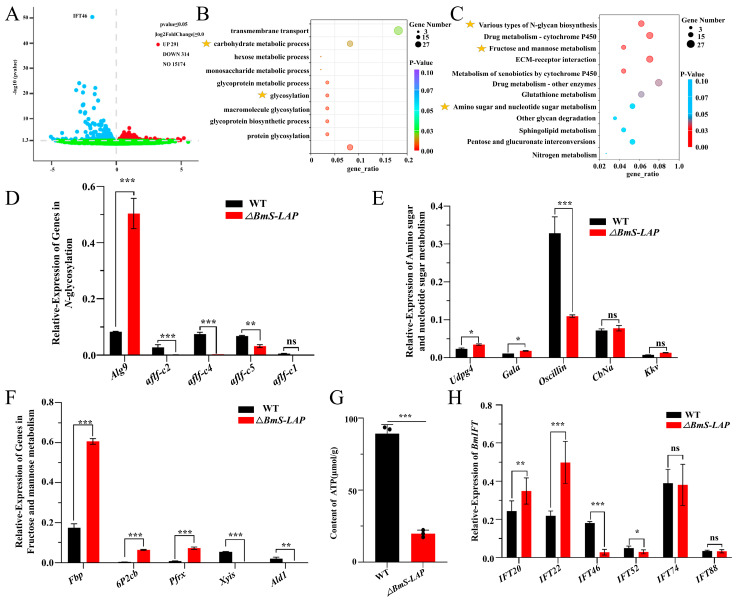
**Transcriptomic analyses of *BmS-LAP* mutants.** (**A**) Dysregulated genes in *ΔBmS-LAP* animals individuals. Scatter plot analysis revealed differentially expressed genes between mutants and WT controls, with red, blue and green points representing up-regulated, down-regulated and non-changing genes, respectively. (**B**) Gene Ontology (GO) terms significantly enriched in differentially expressed genes between *BmS-LAP* mutants and wild-type (WT). (**C**) Top enriched KEGG pathways. (**D**) Relative expression of genes in N-glycosylation pathway. (**E**) Relative expression of genes in Amino sugar and nucleotide sugar metabolism pathway. (**F**) Relative expression of genes in Fructose and mannose metabolism pathway. (**G**) Content of ATP in testis of WT and *ΔBmS-LAP* silkworm at wandering stage. (**H**) Relative expression of *BmIFT* genes. Abbreviations of genes are shown in Table S2. Yellow five-pointed stars indicate the pathways we are focusing on. “ns”, “*”, “**” and “***” represents non-significant difference, “*p* < 0.05”, “*p* < 0.01” and “*p* < 0.001” respectively.

To further explore the molecular mechanisms of the *∆BmS-LAP* spermatogenesis defects, RNA-seq analysis was performed using the mixed testes samples from three individual *ΔBmS-LAP* animals and three individual WT animals at wandering stage. In total, we identified 605 significantly differentially expressed genes (DEGs), among which 291 genes were up-regulated and 314 were down-regulated in *ΔBmS-LAP* testes (Figure 6A). GO and KEGG enrichment analyses revealed that DEGs were significantly enriched in energy metabolism related pathways, such as carbohydrate metabolic processes, N-glycan biosynthesis, fructose and mannose metabolism, and amino sugar and nucleotide sugar metabolism (Figure 6B,C). Consistent with the RNA-seq results, qRT-PCR analysis confirmed that many genes within these pathways were significantly dysregulated in mutant testes (Figure 6D–F). Additionally, we detected a significantly decreased ATP content in adult mutant sperm relative to WT controls (Figure 6G). These findings suggested that *BmS-LAP* deficiency induces dysregulation of energy metabolism in sperm. IFT complex B (IFT-B) mediates anterograde transport of flagellar components from the cell body to the assembly site, and its dysfunction is known to cause defects in sperm flagellum formation [54,55,56,57]. RNA-seq analysis also indicated significantly reduced expression of *intraflagellar transport 46* (*IFT46*) in mutant testes (Figure 6A). Given the shortened eupyrene sperm bundles observed in mutants and the known role of IFT proteins in sperm tail assembly, we next examined the expression of other genes related to the IFT-B complex. The results showed that the expression of *IFT46* and *IFT52* was significantly downregulated, while *IFT20* and *IFT22* were upregulated, indicating the dysregulation of the IFT-B complex (Figure 6H). In summary, the data above suggested that the eupyrene spermatogenesis defects of *BmS-LAP* mutants were related to the dysregulation of energy metabolism pathways and expression of IFT genes.

## 4. Discussion

LAPs represent a conserved family of metalloproteinases that function by cleaving the N-terminal residues of peptides and proteins. They are almost universally distributed across all organisms, spanning from bacteria to humans [27,28,29,30]. In addition to their broad-spectrum housekeeping functions, extensive moonlighting roles of LAPs have also been elucidated. For instance, multiple studies have demonstrated that plant LAPs function as molecular chaperones under abiotic stresses like heat or drought, by preventing protein aggregation and aiding refolding [58]. In mammals, LAPs present in the ocular lens may contribute to cataract development due to their impaired ability to process lens crystallins. While the functions of LAPs in stress adaptation and pathology have been established, their role in spermatogenesis has received less attention [59]. Proteomic evidence, however, confirms their presence as integral components of spermatozoa across species [50]. Yet, the precise contribution of these LAPs to sperm formation and function requires further investigation. S-LAPs were initially discovered and described in *D. melanogaster* through sperm proteome analysis [34,35]. Later studies have demonstrated that LAPs are implicated in male fertility in dipterans such as *D. melanogaster* and *Aedes aegypti* [15,33]. Although S-LAPs have been identified in lepidopteran sperm proteomes, their potential role in reproduction remains largely unexplored [36]. In addition, S-LAPs have an interesting functional and evolutionary history, as many appear to have lost catalytic activity. However, whether this loss is universal remains unclear [60]. In the current study, we performed functional analysis of *S-LAP* and confirmed its critical roles in male fertility of *B. mori*.

Lepidopterans undergo a distinctive dimorphic spermatogenesis, giving rise to two distinct coexisting types of sperm: the nucleated eupyrene sperm and the anucleate apyrene sperm [53]. However, the regulatory mechanisms that determine the distinct cell fates of eupyrene versus apyrene sperm remain largely elusive. In this study, we demonstrate the essential role of *BmS-LAP* in eupyrene sperm maturation and male fertility in *B*. *mori*, thereby providing new insight into the mechanism of dimorphic spermatogenesis. *BmS-LAP* exhibits a testis-specific expression pattern, which is consistent with the expression profiles of the eight *S-LAPs* in *D. melanogaster*. Our data demonstrated that while apyrene sperm development proceeded normally, *BmS-LAP* depletion led to severe defects exclusively in eupyrene sperm, disrupting nuclear positioning and flagellar elongation. In contrast to the paracrystalline and mitochondrial defects caused by *S-LAPs* deficiency in *D*. *melanogaster*, our analysis in *B. mori* identified a specific failure in eupyrene sperm bundle elongation and nuclear positioning. The mislocalized nuclei and shortened flagella in mutant eupyrene sperm implicate *BmS-LAP* in the cytoskeletal reorganization and nucleocytoplasmic remodeling that are essential for late spermiogenesis.

Spermiogenesis is a developmental stage characterized by substantial protein biosynthesis as well as demanding energy consumption [61,62,63,64]. Our transcriptomic analysis revealed that differentially expressed genes (DEGs) in the mutant were significantly enriched in pathways related to glycosylation and carbohydrate metabolism, suggesting a profound impact on energy metabolism. Consistent with this, ATP content was significantly reduced in mutant sperm. The significant enrichment of pathways for N-glycan biosynthesis, amino sugar metabolism, and fructose/mannose metabolism suggests that *BmS-LAP* deficiency may disrupt both energy substrate provisioning and key post-translational modifications, thereby compromising processes essential for spermiogenesis. Glycosylation is a well-established, fundamental post-translational modification that governs protein folding, stability, and function across diverse biological systems [65,66,67]. Our data suggested that *BmS-LAP* disruption, causing an N-glycosylation defect marked by the downregulation of *Alg9* and key fucosyltransferases (*BmAflf-c2*, -*c4*, -*c5*), could directly impair the function of membrane proteins and ion channels necessary for flagellar assembly and sperm motility. Our study suggests a potential link between alterations in glycosylation pathways and spermatogenesis defects in *B. mori*, expanding our understanding of the metabolic regulation underlying sperm development.

Intraflagellar transport is a conserved process responsible for the assembly and maintenance of cilia and flagella by transporting cargo proteins along the axonemal microtubules [54,68]. In our data, the downregulation of *intraflagellar transport* (*IFT*) genes, particularly *IFT46*, provides a potential explanation for the defective flagellar elongation, which is phenotypically manifested as shortened sperm flagella that align with the compromised expression of these core IFT components. In accordance with the speculation, the disruption of axoneme assembly is a core cause of defective sperm flagella across species. In mice, mutations in *IFT88*, *IFT20*, or *IFT27* result in shortened and severely coiled tails, leading to abnormal sperm morphology [55,56,57]. Similarly, in humans, impairments in *IFT140* or *IFT172* result in sperm with short, bent tails and a loss of motility, leading to male infertility [69,70,71]. Conversely, while *Drosophila IFT52* mutants exhibit severe defects in cilia-related sensory behaviors, they can still produce sperm with normal motility, supporting a dispensable role for *IFT52* in sperm flagella formation [72,73].

## 5. Conclusions

In conclusion, this study establishes *BmS-LAP* as a critical regulator of eupyrene sperm maturation in *B. mori*. Our work not only elucidates a key gene function in lepidopteran reproduction but also suggests a potential nexus between metabolic regulation and cytoskeletal morphogenesis during sperm development. In *D. melanogaster*, S-Lap proteins function independently of their canonical aminopeptidase activity [31]. Thus, future work should aim to clarify the specific protein substrates of *BmS-LAP* and determine whether it functions via its leucine aminopeptidase activity.

## Data Availability

The original contributions presented in the study are included in the article. Further inquiries can be directed to the corresponding author. The RNA-seq raw data in this study have been deposited in in the NCBI SRA database with accession PRJNA1445539 (https://www.ncbi.nlm.nih.gov/bioproject/PRJNA1445539/), accessed on 31 March 2026.

## Supplementary Materials

The following supporting information can be downloaded at: https://www.mdpi.com/article/10.3390/insects17040389/s1. Table S1. Primers used in this study. Table S2. Abbreviation of genes.
