# Supplementary material for: The Development of Eupyrene Sperm Is Dependent on Sperm-Leucylaminopeptidase in Bombyx mori
Source: Insects. 2026 Apr 3;17(4):389. doi: 10.3390/insects17040389 (PMC13115824; doi:10.3390/insects17040389)
Supplement: Supplementary file 1 [file insects-17-00389-s001.zip › insects-4194857-supplementary.pdf]

Table S1. Primers used in this study.

Primer name	Primer sequence(5'to3')	Purpose
RP49-F	TCCAACAAGAAGACCCGTCA	qRT-PCR
RP49-R	GCTCTTTCCACGATCAGCTT	qRT-PCR
HindIII-F	CGAGGTCGACGGTATCGATAAGGTTATG TAGTACACATTGTTGTA	Plasmid construction
HindIII-R	TTTCAAGTGATAACGGACTAGCCTTATTT CTTGCTATTTCTAGCTCTAAAAC	Plasmid construction
Overlap-F	GCTAGCCATTGACTCCGCGGATCGAGGTT ATGTAGTACACATTGT	Plasmid construction
Overlap-R	ACAATGTGTACTACATAACCTCGATCCGC GGAGTCAATGGCTAGC	Plasmid construction
LAP-sg1-F	CTGATCTCAACGTAGAGCGGGTTTTAGA GCTAGAAATAGCAAGTT	Plasmid construction
LAP-sg1-R	CCGCTCTACGTTGAGATCAGACTTGTAGA GCACGATATTTTGTAT	Plasmid construction
LAP-sg2-F	GGATTTGGCGAATGCCATTAGTTTTAGAG CTAGAAATAGCAAGTT	Plasmid construction
LAP-sg2-R	TAATGGCATTCGCCAAATCCACTTGTAGA GCACGATATTTTGTAT	Plasmid construction
LAP-seq-F	CTTTGGAAATGCAGAAGCCG	Mutation detection
LAP-seq-R	GCGGCGAAGATATGGGAAAT	Mutation detection
LAP-qPCR-F	GGAGCAGGTGAGAAAACTCG	qRT-PCR
LAP-qPCR-R	TGGCTAATATGCATCCAGCA	qRT-PCR
aflf-c1-qPCR-F	ACCAAGTTTCTGCCACCTGT	qRT-PCR
aflf-c1-qPCR-R	TCCAGCTGTGGTAGCCATTG	qRT-PCR

aflf-c2-qPCR-F	GGTAAACTGACCTGCCCGAA	qRT-PCR
aflf-c2-qPCR-R	TGAGTAATTAGCGCCGCCAT	qRT-PCR
aflf-c4-qPCR-F	TTGGAAGCCAACGCAACAAC	qRT-PCR
aflf-c4-qPCR-R	GCATCAAAGTGTTCAGTGCCT	qRT-PCR
aflf-c5-qPCR-F	AGGAACCATTACGCCGTCAG	qRT-PCR
aflf-c5-qPCR-R	TCGGTCCTCGTATTCAGGGT	qRT-PCR
Alg9-qPCR-F	CGCTGACCGTACGACAAAGA	qRT-PCR
Alg9-qPCR-R	TGACAATGTAATCTCCTTCGCT	qRT-PCR
udpg4-F	AGTGCCACTCAAGTACGTCG	qRT-PCR
udpg4-R	TGTCTGCCATCGCCAGAAAT	qRT-PCR
gala-F	GGCAAAAAGGACCACGCATT	qRT-PCR
gala-R	GCTTCTTGACACTGAGCCCT	qRT-PCR
Oscillin-F	GTATGTGGGTCTTCCCCGTG	qRT-PCR
Oscillin-R	CTAAATCGGATGCGTTGCCG	qRT-PCR
xyis-F	GCTTCCCGGAGGTATCAACC	qRT-PCR
xyis -R	CCCTGGCTCTTCCTGCAAAT	qRT-PCR
Pfrx-F	GCAGGACAAGGAAGAAGCCA	qRT-PCR
Pfrx-R	ATCCCCATTCGTTTCGCCAT	qRT-PCR
ald1-F	ACCCCCTCGTACCAAGCTAT	qRT-PCR
ald1-R	TACACGTGGTGGTCGTTCAG	qRT-PCR
6p2cb-F	CGGCCATCAAGCTGTACTGA	qRT-PCR
6p2cb-R	TTGTTCCGCTCTCACCTTGG	qRT-PCR
fbp-F	CTGGACGTCCTTTCCAACGA	qRT-PCR
fbp-R	AGAGGGTCGAAACACACCAC	qRT-PCR

cbNa-F	TGGAGATGGACGTGCGAAAA	qRT-PCR
cbNa-R	GCCAGAGGAGACCGTAATCG	qRT-PCR
Kkv-F	TGACGATGAACTCACACCCC	qRT-PCR
Kkv-R	TGAAGGTGACCAGGTAAGCG	qRT-PCR
IFT20-F	TGGCTGCAATAGGAGCAATGA	qRT-PCR
IFT20-R	TGCAATTTCAGCTGTTCGGA	qRT-PCR
IFT22-F	GGTCCCTCTGAAAGTGGCAA	qRT-PCR
IFT22-R	GAGTAGGCTTCGGGTTTCCC	qRT-PCR
IFT46-F	ACGAAGGTTACCAAGGCAGG	qRT-PCR
IFT46-R	GTTCCATTTCTCCGCTATCAGAA	qRT-PCR
IFT52-F	TATAGGGCACAGCCACCTCT	qRT-PCR
IFT52-R	GAACCGACATTGGATCGGGA	qRT-PCR
IFT74-F	CGCGCGTCATGAAAGAAGAG	qRT-PCR
IFT74-R	CGGTAACAGCACTCTCGGTT	qRT-PCR
IFT88-F	CCATGCAATGCTGGATGTCG	qRT-PCR
IFT88-R	TGCCAGACATCGTTCAGCTT	qRT-PCR

Table S2. Abbreviation of genes.

abbreviation	full name
Fbp	fructose-1,6-bisphosphatase
6p2cb	6-phosphofructo-2-kinase/fructose-2,6-bisphosphatase
Pfrx	6-phosphofructo-2-kinase/fructose-2,6-biphosphatase
Xyis	xylose isomerase
Ald1	fructose-bisphosphate aldolase
Udpg4	UDP-glucose 4-epimerase
Gala	galactokinase
Kkv	hyaluronan synthase-like protein kkv
CbNa	chitooligosaccharidolytic beta-N-acetylglucosaminidase
Oscillin	glucosamine-6-phosphate isomerase Oscillin
Alg9	alpha-1,2-mannosyltransferase Alg9
aflf-c2	alpha-(1,3)-fucosyltransferase C2
aflf-c4	alpha-(1,3)-fucosyltransferase C4
aflf-c5	alpha-(1,3)-fucosyltransferase C5
aflf-c1	alpha-(1,3)-fucosyltransferase C1
